# Emerging Roles of Matricellular Proteins in Systemic Sclerosis

**DOI:** 10.3390/ijms21134776

**Published:** 2020-07-06

**Authors:** Daniel Feng, Casimiro Gerarduzzi

**Affiliations:** 1 Département de Pharmacologie et Physiologie, Faculté de Médecine, Université de Montréal, Montréal, QC H3T 1J4, Canada; daniel.feng@umontreal.ca; 2 Centre de recherche de l’Hôpital Maisonneuve-Rosemont, Faculté de Médecine, Centre affilié à l’Université de Montréal, Montréal, QC H1T 2M4, Canada; 3 Département de Médecine, Faculté de Médecine, Université de Montréal, Montréal, QC H3T 1J4, Canada

**Keywords:** systemic sclerosis, fibrosis, extracellular matrix, matricellular proteins, myofibroblasts, biomarkers/therapeutics

## Abstract

Systemic sclerosis is a rare chronic heterogenous disease that involves inflammation and vasculopathy, and converges in end-stage development of multisystem tissue fibrosis. The loss of tight spatial distribution and temporal expression of proteins in the extracellular matrix (ECM) leads to progressive organ stiffening, which is a hallmark of fibrotic disease. A group of nonstructural matrix proteins, known as matricellular proteins (MCPs) are implicated in dysregulated processes that drive fibrosis such as ECM remodeling and various cellular behaviors. Accordingly, MCPs have been described in the context of fibrosis in sclerosis (SSc) as predictive disease biomarkers and regulators of ECM synthesis, with promising therapeutic potential. In this present review, an informative summary of major MCPs is presented highlighting their clear correlations to SSc- fibrosis.

## 1. Introduction

Systemic sclerosis (SSc) is a rare idiopathic disease that presents as a trifecta of compounding chronic abnormalities driven by autoimmunity, vasculopathy, and systemic tissue fibrosis [1]. SSc carries considerable phenotypic heterogeneity, which consequently leads to the poorly understood complex interplay amongst the dysregulated systems. As it affects major internal organ systems, such as the lungs and heart during disease progression, the fibrotic tissue decreases survival probability, and accounts for a large proportion of SSc-related deaths due to organ failure [2]. Optimistically, reports of survival rates amongst various global SSc cohorts published within the last 5 years have shown a steady upwards trend to >90% and 80–90% in 5- and 10- year survival rates, respectively [3,4,5,6,7]. Innovations in earlier detection and improved stratification of SSc subtypes through advances in genomic and metabolic screening have led to consistent improvements, and has enabled healthcare professionals to make informed and timely decisions on appropriate therapeutic interventions [8,9,10,11]. 

The burden of organ fibrosis in SSc is the driving motivation behind a large number of novel research studies, with nearly double the amount of studies published on NCBI PubMed within the last decade (2470 publications listed under the key search terms of “systemic sclerosis” + “fibrosis”) compared to previous years (1484 publications). Fibrosis is a deregulated repair process defined by the progressive stiffening and scarring of tissue due to the excessive deposition of extracellular matrix (ECM) components, causing loss of native organ architecture and impairment of homeostatic function [12,13]. Fibrogenesis of visceral organs pose significant stress on patient quality of life. In fact, the destructive scarring of the lungs is the leading cause of mortality in both diffuse (dcSSc) and limited (lcSSc) cutaneous SSc patient populations [14,15,16]. Apparent histological and physiological similarities exist amongst different organs in end-stage fibrotic disease; thus, leveraging this fact allows fibrosis research to discover conserved pathological mechanisms regardless of etiology. 

At the root of fibrotic disorders is a dysregulated ECM caused by aberrant accumulation of structural matrix proteins, primarily collagens types I, III, and IV, fibronectin, fibrin, and elastins [17,18]. Consensus in the literature points to activated fibroblasts, known as myofibroblasts, as the primary effector cell type responsible for the overproduction and management of the various ECM components leading to elevated mechanical stress, tension, and reduced plasticity of the ECM network [18]. Myofibroblasts are not normally found in healthy connective tissue but emerge in circumstances of acute insults, chronic inflammation, and repeated tissue injury [19]. Myofibroblasts are shown to be vastly heterogenous in lineage and arise from not only differentiation of the reparative resident fibroblasts, but also circulating progenitor cells such as fibrocytes and pericytes that collectively contribute to the stiffening of the matrix environment [19,20,21]. After an injury has been resolved, the natural mechanisms for clearing myofibroblasts include apoptosis, dedifferentiation, and senescence, which happen to be deregulated in chronic injuries due to the prosurvival signals and biomechanical cues; hence, supporting their persistent activation and hindering fibrosis resolution [19]. 

Several key signaling pathways allow myofibroblasts to destructively release excessive ECM proteins while simultaneously evading their depopulation. TGFβ is a pleiotropic cytokine with broad downstream signaling effects that have been extensively reviewed in SSc pathogenesis for its roles in autoimmunity, vasculopathy, myofibroblast differentiation, and ECM synthesis [19,20,22,23]. The TGFβ-induced phosphorylation of SMAD proteins (SMAD1 and SMAD2/3) underlies pathological fibrosis by stimulating both fibroblast to myofibroblast differentiation and activation of profibrotic genes, largely through α-smooth muscle actin (αSMA) and collagen upregulation, respectively [24,25,26]. Moreover, canonical Wnt-signaling in myofibroblasts via its ligands Wnt1, Wnt-3a, and Wnt-10b have been shown to upregulate expression of ECM encoding genes under ECM restructuring conditions [20,27,28]. Collectively, these pathways along with many others described in the literature empower myofibroblasts to be the driving force in fibrotic pathologies. 

In addition to the aberrant biochemical signaling from myofibroblasts, the delicate balance in biomechanical forces of the ECM is disrupted. The ECM is increasingly being appreciated for its effect on cells during the processing of its structure to meet organ-specific demands. In recent years, we have been learning that one way by which this is achieved is through matricellular proteins (MCPs), which are a group of matrix proteins with nonstructural function and expressed in a context-dependent manner [29]. MCPs have also been emerging for their profound effects on myofibroblasts, such as their differentiation, recruitment, collagen synthesis, and apoptotic evasion under fibrotic conditions [19,20,30,31]. 

## 2. Function of MCPs

In contrast to the classical structural proteins of the ECM, MCPs function distinctly as nonstructural extracellular signaling molecules that mediate the dynamic biochemical and biomechanical status of the ECM to the surrounding cell populations. Basal expression levels of MCPs are typically quite low after development, but are transiently re-expressed for their multitude of functions during early tissue remodeling processes. One context in which the role of MCPs is best exemplified is during wound repair, whereby tight temporal and spatial re-expression of MCPs are required for timely resolution of an injury. It may do so by contributing to the transformation of fibroblasts into myofibroblasts, and triggering intracellular events to stimulate proliferation, cell migration, and invasion of myofibroblasts to the injury site. Furthermore, their expression is associated with ECM remodeling processes such as stimulating synthesis of matrix proteins (i.e. collagen, fibronectin, and elastins) and proteolytic degradation of the ECM to regain the structural integrity lost after tissue damage. MCPs can also directly interact with matrix components through their distinct functional domains, allowing them to anchor to the ECM network [31]. These evolutionary conserved functional domains have been used to classify and group like MCPs together into six unique families: Centralized Coordination Network (CCN), Thrombospondin (THBS), Secreted Protein Acidic and Rich in Cysteine (SPARC), Tenascin (TN), Small Integrin-Binding Ligand N-Linked Glycoprotein (SIBLING), and γ-carboxyglutamate (Gla)-containing proteins [32].

As extracellularly localized molecules, MCPs are capable of communicating with surrounding cells by engaging different cell surface receptors to elicit an intracellular response (Table 1). One of the most common interactions is the binding of MCPs with integrins, which are cell surface αβ heterodimeric receptors. Integrins are composed from one of 18 α subunits and 8 β subunits, which form unique combinations to recognize and bind different ECM molecules. Integrin-mediated signaling enables MCPs to physically bridge the ECM with the cellular cytoskeleton and relay dynamically changing ECM structures to the cell. This occurs through a process called mechanotransduction, whereby extracellular physical changes that alter the plasticity (i.e. stiffening, degradation) of the ECM can be transformed intracellularly to induce chemical signaling cascades [33,34]. In addition to integrins, MCPs can also bind to membrane-bound heparan sulfate containing proteoglycans such as syndecans, which can act independently or as coreceptors to integrins and growth factor receptors to modulate cell–matrix interactions [35,36]. For example, syndecan–MCP and syndecan–integrin interactions are important for focal adhesion formation during cell adhesion and spreading, as well as the activation of focal adhesion kinases to induce actin reorganization [37]. MCP binding to cell surface calreticulin is also known to mediate assembly and disassembly of focal adhesions by activation of signaling pathways such as PI3K and ERK [38]. The presence of divalent ions (i.e. Ca^2+^ and Zn^2+^) as well as binding of coreceptors, such as LDL-receptor-related proteins, can additionally enhance calreticulin-MCP functions. Recognition of cell membrane receptors CD36, CD44, and CD47, predominately by thrombospondins, in contexts of cell senescence, cancer, and immune regulation further emphasize the breadth of MCP receptor binding partners [39,40,41]. Other unique interactions of MCPs include tyrosine kinase receptor TrkA, Wnt-receptors LRP1 and LRP6, Annexin II, EGFR, and TLR4 (Table 1) [36,42,43,44].

Intracellular signal transduction is also mediated by MCPs through modulation of soluble matrix cytokines and growth factors. Importantly, their interaction with pleiotropic signaling molecules such as TGFβ, VEGF, Wnt, and EGF confer broad biological functions such as stimulating ECM production and remodeling, angiogenesis, and regulating cellular differentiation [36]. To regulate these functions, MCPs can affect conformational changes in the ECM to release embedded growth factors and convert latent forms of biomolecules in the ECM [36]. 

Owing to their dysregulation of diverse functions in human pathology, MCPs have certainly not been ignored in disease but perhaps their potential has not been entirely realized either. MCPs are largely recognized in fibrotic settings where excessive ECM production is central to disease progression. As fibrosis is one of the central axes of disease in SSc, this review aims to bring attention to the current insights of MCPs in SSc by discussing the burgeoning roles of the most well-known MCP families. 

## 3. CCN Family

The CCN family of MCPs are composed of six proteins designated CCN1 to CCN6. Of greatest structural significance to this family of proteins is their unique tetramodular organization that confer vast biological properties acting both independently and/or complementary to one another [57]. Loss of tight spatial-temporal regulation of CCN proteins in wound healing triggers a maladaptive repair program affecting processes of inflammation, proliferation, and tissue remodeling that can lead to development of fibrosis-related pathologies such as SSc [58,59,60]. Amongst the CCN family, CCN1 and CCN2 make up the majority of MCPs studied in SSc research while CCN3-6 are less understood but have emerging functions. 

CCN1 (alternatively cysteine-rich angiogenic protein 61, CYR61) is a well characterized MCP with generally profibrotic and angiogenic influence [61]. Post developmental expression of CCN1, like many MCPs is observed during tissue damage and disease; however, circulating levels of CCN1 in serum samples of SSc patients have been inconsistently reported in the literature. Lin et al. report CCN1 to be significantly elevated in SSc from their analysis of several autoimmune diseases [62], while other reports found no difference in plasma CCN1 in both lcSSc and dcSSc patients versus healthy controls [63,64]. Protein levels of CCN1 from dermal fibroblasts of lesioned skin also reveal conflicting expression patterns. Comparable expression between healthy and SSc-derived fibroblasts was reported by Tsou et al., while fibroblasts from a small cohort of patients with early onset (<2 years) dcSSc found CCN1 to be overexpressed in comparison to healthy matched controls [63,65].

CCN1 has also been conflicting for its mechanistic role in SSc, showing both pro- and antifibrotic effects. Using an in vivo model of bleomycin-induced skin scleroderma in mice, targeted knockout of CCN1 in fibroblasts limited type 1 collagen accumulation with reduced skin thickness [65]. Alternatively, CCN1 overexpression in isolated dermal fibroblasts cultured from skin biopsies of dcSSc patients suggest an antifibrotic role due to the downregulated expression of fibrotic genes *COL1A1* and *ACTA2*, and consequently, slower cell migration and diminished gel contractile strength [63]. A mechanistic explanation by Tsou et al. propose a twofold rationale for the attenuation of fibrogenesis by CCN1 overexpression in these dcSSc fibroblasts. They describe an impairment of TGFβ receptor-II and SMAD2/3 phosphorylation, which is further compounded by elevated levels of reactive oxygen species activating various senescent signaling cascades (p38 MAPK, p53, and p16/phospho-pRB) [63].

CCN2 (alternatively connective tissue growth factor, CTGF) is the most well-known CCN family member. Accumulated evidence has proved its essential role in mediating organ fibrosis of different etiologies [66,67]. In SSc, overexpression of CCN2 is a systemic phenomenon with elevated mRNA and protein levels detected not only in areas of lesioned fibrotic skin but also in the dermal interstitial fluid and serum of SSc patients [67,68,69,70]. CCN2 is also upregulated in the epidermal layer of SSc skin and in epidermis-conditioned media of SSc patients when compared to healthy controls [71]. Soluble CCN2 is also shown to concentrate at the epidermis–dermis junction and blood vessels, supporting the notion that it is a crucial factor in maintaining epidermal fibrosis in SSc. Mechanistically, CCN2 can be induced by TGFβ signaling in a SMAD-dependent fashion but is also upregulated in response to biomechanical changes in the ECM during tissue stiffening in fibrosis [72,73]. Contractility of SSc lung fibroblasts was found to be greater with the addition of recombinant CCN2 protein, which correlated with αSMA expression [74]. Moreover, enhanced expression of CCN2 by scleroderma fibroblasts is shown to promote collagen gel contraction that is dependent on transcription factors known to communicate mechanical adaptions and cues from the ECM [75]. In a separate study, CCN2 was further shown to be upregulated in SSc fibroblasts stimulated with TGFβ and seeded on rigid polyacrylamide gels that recapitulated stiffness of sclerotic dermal tissue [76]. Overall, the mechanoregulatory roles of CCN2 is a growing topic of interest in scleroderma with various pieces of evidence supporting the notion that it is a crucial factor in maintaining epidermal fibrosis in SSc.

CCN3 (alternatively nephroblastoma overexpressed, Nov) is regarded as an antagonist to CCN2 in fibrosis [59]. Its antifibrotic effect has been suggested to work via repression of CCN2, possibly by non-SMAD-dependent signaling pathways or affecting gene expression of other CCN members like CCN4 [59,77,78]. Microarray analysis of both lcSSc and late dcSSc skin biopsies reveal no significant difference in CCN3 expression, while a modest upregulation is observed in early dcSSc skin [79]. In the tight-skin (*Tsk1/+*) mice model of SSc, overexpression of CCN3 was found to repress fibrillin-1 and elastin assembly by antagonistically modulating TGFβ and Wnt-regulated gene expression [79].

CCN4-6 are a set of three Wnt-pathway target genes; hence, they are alternatively abbreviated as WNT1-inducible-signaling pathway protein 1–3 (Wisp-1–3), respectively. Presently, little is known regarding this trio of CCN proteins in the context of SSc, but further studies could potentially discover an association since they are known for their roles in non-SSc-related fibrotic disorders [80,81]. From biopsies of SSc skin, CCN4 and CCN6 expression was not detected at the mRNA level, while CCN5 expression was reported to be downregulated when compared to normal patients [60,82]. In a different patient cohort, microarray data revealed CCN4 to be strongly associated with the profibrotic transcription factor Egr-1, which is also a downstream target of TGFβ in primary human fibroblasts [79,83]. CCN4 also promotes alveolar type II cell proliferation, matrix metalloprotease (MMP) secretion, and fibroblast matrix secretion in interstitial lung disease, each a common fibrotic manifestation in SSc [84,85]. Although CCN4-6 have not been directly studied for their mechanisms in SSc, it is very likely that their known interaction with Wnt signaling may be involved since the pathway is prominently altered in SSc.

## 4. Thrombospondin Family

The thrombospondins are matrix-secreted glycoproteins that consist of five family members sharing a conserved signature domain (THBS-1-5) [86]. THBS proteins are organized in a modular fashion as trimeric (THBS-1,2) and pentameric (THBS-3-5) oligomer structures. THBS proteins interact with an array of matrix molecules to activate various pathways implicated in tissue repair, ECM remodeling and mineralization, and angiogenesis [87,88,89]. Research on THBS proteins in SSc suggest an overall profibrotic role for THBS-1,2,4, and -5, while THBS-3 has not yet been described.

THBS-1 is a widely recognized profibrotic MCP found to be overexpressed in fibrosis of various organs, such as in the kidneys and lungs [90]. In SSc, THBS-1 is a predictive longitudinal biomarker of lesioned skin development, as proposed originally by the Lafyatis group in their four-gene biomarker set for SSc [8,91,92]. Its emergence as a reliable marker of fibrotic progression has been leveraged as a comarker for monitoring disease development and response to therapeutics in SSc [92,93,94,95]. Several recent and on-going clinical trials also show THBS-1 to parallel changes in modified Rodnan skin scores (MRSS) from skin biopsies, which is considered the gold standard for assessing improvement in skin thickness in response to therapeutics [93,94,95]. In vitro experiments using dcSSc dermal fibroblasts by Chen et al. have also demonstrated that THBS-1 expression is induced by mechanical force loading, and is an endogenous activator of latent TGFβ during matrix contraction to enhance contractile activity of pathological SSc fibroblasts [96]. In the same study, expression of THBS-1 in SSc fibroblasts was also limited by inhibiting the PDGF pathway with pharmacologic agents that antagonized MEK/ERK pathway activation. Finally, a selective inhibitor of THBS-1 has been recently shown to serve as a novel hypertrophic scar treatment [97]. Upregulated THBS-1 in scarred skin was antagonized by a LSKL (leucine-serine-lysine-leucine) peptide that augmented fibroblast contractility and attenuated release of type I collagen and αSMA accumulation [97]. Taken together, inhibiting THBS-1 may be effective in managing multiple symptoms of SSc at different stages of disease progression.

Pioneer work on MCPs by the Bornstein group established a role of THBS-2 as a crucial modulator of collagen fibrillogenesis in ECM assembly and wound repair, as well as influencing fibroblast adhesion and contraction [98,99]. In the case of SSc patients, THBS-2 is elevated in skin biopsy sections and the serum of those containing pitting scars and/or ulcers, when compared to healthy controls [100]. In vitro analysis of isolated SSc dermal fibroblasts found THBS-2 mRNA to be downregulated, but a significant extracellular accumulation of THBS-2 was observed in the culture medium and immunohistological staining of biopsy sections. Moreover, silencing of THBS-2 by siRNA downregulated type I collagen synthesis in SSc fibroblasts. Taken together, the extracellular presence of THBS-2 contributes to the upregulation and buildup of type I collagen, which could then promote a profibrotic environment in scleroderma.

THBS-5, or alternatively cartilage oligomeric matrix protein (COMP), is constitutively expressed in the ECM of connective tissues and functions as a catalyst for collagen fibrillogenesis [101,102]. THBS-5 has emerged as a key component of homeostatic matrix remodeling through its interaction with structural proteins in the ECM (collagen, fibronectin, and matrilin), growth factors (TGFβ and BMP-2,4,7), and proteases (MMP-13 and ADAMTS-4,7,12) via distinct portions of its conserved signature domain [103,104,105]. In correlation to these profibrotic factors, THBS-5 overexpression was observed and involved in various fibroproliferative pathologies. THBS-5 was also a part of the Lafyatis SSc four-gene biomarker set, and detection of serum THBS-5 in early diagnosed SSc patients has been shown to be predictive of pulmonary fibrosis onset and mortality [92,106]. Moreover, serum THBS-5 was shown to parallel progressive changes in total skin thickness as measured using standardized methods such as MRSS and high frequency ultrasound [107]. Accumulation of THBS-5 in the dermis is spatially heterogenous and largely secreted by SSc fibroblasts in response to autocrine TGFβ stimulation, which leads to matrix stiffening and skin tightening by excessive collagen deposition [108].

Although very little is known regarding THBS-4 in SSc, its fibrotic properties are largely described in the context of cardiac tissue remodeling where mice lacking THBS-4 develop interstitial fibrosis by inducing increased deposition of collagen I, II, III, and V [109]. Recent reports also suggest that the upregulation of THBS-4 expression by TGFβ is mediated specifically by SMAD3 signaling to exert proangiogenic effects [110]. Therefore, understanding THBS-4 in other fibrotic pathologies could help to better understand its role in SSc. Interestingly, the only documented case of THBS-4 in SSc was recently published by Moon et al. The group applied a method of machine learning to mine a gene expression compendium of SSc patients and identified the genes *THBS-1*, *THBS-4*, *THBS-5*, *FN1*, and *TN-C* to best represent changes in the severity of skin fibrosis [111]. This representative gene set displayed the strongest correlation to MRSS fibrotic scores, which was likely due to their ability to drive skin fibrosis.

Members of the THBS family are well documented for their role in different organ fibrotic mechanisms, though those implicated in driving the pathology of SSc are less understood. While having clinical relevance as serological biomarkers for the fibrotic burden in SSc, THBS proteins, particularly THBS-3, present an area of opportunistic research to better understand their contributing roles in SSc fibrosis, which can be potentially aided by drawing parallels with their roles in other fibrotic organ models.

## 5. SPARC Family

The SPARC family is made up of the eight proteins, SPARC, SPARCL1, SMOC-1-2, SPOCK-1-3, and FSTL1, all of which share a follistatin-like domain and an extracellular calcium-binding domain [112]. SPARC family members are broad functioning matrix modulating proteins expressed in connective tissues and have been associated with a large spectrum of human pathologies, from maladaptive tissue repair to cancer and autoimmune disorders [52,112,113,114]. In SSc, particular attention has been given to SPARC and FSTL1, while the other family members are very understudied.

SPARC is among one of the first MCPs described and has since been an invaluable template in dissecting the role of MCPs in disease. SPARC overexpression is a common theme in various fibrotic pathologies, including SSc where elevated circulating SPARC is observed in SSc patients compared to matched counterparts [115,116]. At the cellular level, RT-PCR analysis of fibroblasts isolated from lesioned skin of dcSSc patients show an upregulation of fibrotic ECM genes *COL1A2* and *COL3A1*, as well as fibrotic MCP genes *SPARC* and *CCN2* when compared to healthy controls [117]. Silencing SPARC expression in such fibroblasts by siRNA was sufficient to reduce type I collagen and CCN2 expression. Conversely, SSc dermal fibroblasts treated with exogenous SPARC protein induced protein expression of collagen I, IV, fibronectin, and TGFβ in a dose-dependent manner [118]. Antagonizing TGFβ signaling in these fibroblasts with a TGFβ-R1 inhibitor (galunisertib) blocked phosphorylation of SMAD2, which consequently inhibited the effects of SPARC stimulation. Lastly, an epigenetic relationship has also been reported in Choctaw Native Americans in which several *SPARC* single nucleotide polymorphisms have been associated with a higher prevalence of SSc in this population [119].

FSTL1 has been studied in fewer SSc cases in comparison to SPARC. One particular study has detected elevated levels of circulating FSTL1 in the sera of SSc patients [120]. From a study of epigenetic mechanisms regulated by histone deacetylases (HDAC), *FSTL1*, *CCN1*, and *PVRL2* have been reported for its angiogenic and fibrotic function on dermal endothelial cells (EC) from dcSSc patients [121]. Dual inhibition of HDAC5 in conjunction with either *FSTL1*, *CCN1* or *PVRL2* inhibited angiogenesis, while their overexpression led to an increase in EC tube formation. Despite the limited literature, FSTL1 has promising expectations in SSc when considering the evidence of its involvement in several conventional mechanisms of fibrosis [122,123,124].

## 6. Tenascin Family

The tenascin family is composed of four members, Tenascin-C, -X, -R, and -W (TN-C, TN-X, TN-R and. TN-W). The conserved structure of tenascins includes N-terminal heptad repeats, EGF-like domains, and a series of fibronectin type-III domains that are important for the interaction between cell surface proteins and soluble factors in the ECM [125]. Tenascins are multifunctional MCPs that have been shown to mediate processes of cell adhesion, migration, matrix assembly, profibrotic cytokine upregulation, and myofibroblast differentiation [126,127,128,129]. In SSc research, the primary focus has been on TN-C because its translatable research from related fibrotic pathologies has provided a framework for study, whereas the other family members still remain undercharacterized.

TN-C expression is ubiquitously expressed during embryogenesis but may also display re-expression after fetal development in response to chronic inflammation at sites of wound healing and fibrosis [127]. The increased expression of TN-C in the reticular dermis in scleroderma has been observed through immunohistochemical techniques nearly three decades ago and has also been recently shown in the papillary layer as well [130,131]. TN-C is also a robust marker of SSc pathology not only in dermal skin but its elevated levels are also observed in serum and bronchoalveolar lavage fluid of SSc patients [132,133,134]. TN-C overexpression has been shown to accelerate SSc-related skin and pulmonary fibrosis, by driving tissue inflammation, myofibroblast differentiation, and aberrant deposition of collagen and fibronectin, subsequently leading to dermal stiffening [131,132]. To model SSc-associated acute lung injury, TN-C knockout (TN-C^-/-^) mice were administered intratracheal bleomycin over a period of 14 days to induce fibrosis [131]. In such mice, a reduced fibrotic response was observed with less accumulation of αSMA-positive interstitial myofibroblasts in the lungs, as well as infiltration of inflammatory macrophages and lymphocytes, compared to vehicle-treated wild-type (WT) mice. Reduced collagen buildup and lysyl oxidase expression in the lungs also protected TN-C^-/-^ mice from loss of lung elasticity due to tissue stiffening. Taken together, these findings suggest an overall fibrotic role of TN-C in pulmonary fibrosis. Within the same study, TN-C^-/-^ mice were also studied in conjunction with the *Tsk1*/+ model of noninflammatory associated skin fibrosis. TN-C^-/-^/*Tsk1*/+ mice were generated, and it was found that the absence of TN-C slowed the onset of hypodermal thickening by reducing collagen and αSMA expression, which is characteristic of this model [131].

A mechanism of TN-C-driven fibrogenesis in SSc has been shown in both stromal cells and macrophages through the action of toll-like receptors (TLRs), which are known to be activated in response to tissue injury [131]. Specifically, TN-C is capable of inducing a TLR4-dependent fibrotic response in fibroblasts, resulting in myofibroblast differentiation, collagen gene expression, secretion of Il-6, TGFβ regulation, and upregulation of TLR4 expression itself. Taken together, the aberrant accumulation of both TN-C and TLR4 within SSc lesional tissue is proposed to work co-operatively to create a feed-forward loop that amplifies TLR4 inflammatory and profibrotic signaling.

Although other members of the tenascin family have yet to be studied as extensively as TN-C, few studies have shown emerging roles of TN-X in SSc. Dermal fibroblasts cultured from TN-X-deficient (TN-X^-/-^) mice struggle to deposit sufficient amounts of type I collagen into the ECM, leading to reduced tensile strength and deformability in skin of TN-X^-/-^ mice. Though the deposited collagen fibrils themselves did not exhibit morphological abnormalities, it suggests perhaps a role for TN-X as a regulator of collagen deposition and/or in determining mechanical properties of collagen-rich tissues, but not fibrillogenesis [135,136].

## 7. SIBLING Family

The SIBLING family consists of five members; osteopontin (OPN/SPP1), bone sialoprotein (BSP), dentin sialophosphoprotein (DSPP), dentin matrix protein-1 (DMP1), and matrix extracellular phosphoglycoprotein (MEPE). All SIBLING proteins contain an Arg-Gly-Asp (RGD) integrin-binding motif important for its overall hydrophilic structure and cellular structure [137]. Most SIBLING proteins also activate specific MMPs to mediate processes involved in ECM degradation, and are best described in mineralized tissue such as bone and dentin [137,138]. OPN is the most studied SIBLING protein, and for this reason studies of this family within the context of SSc focuses mainly on OPN.

OPN expression is upregulated during wound repair where it serves to both initiate a proinflammatory response by localizing T-cells and macrophages at the site of injury, and regulate fibroblast behavior and myofibroblast differentiation during tissue remodeling [139]. Dysregulated OPN expression is associated with many fibrotic pathologies, and in vivo animal studies demonstrate that OPN inhibition significantly ameliorates fibrotic symptoms across different organ systems [140,141,142]. The improved fibrotic outcome is the result of several processes such as reduced collagen I/IV accumulation and matrix deposition, decreased local TGFβ activity, and lower myofibroblast proliferation. Unsurprisingly, these insights into the mechanisms of OPN have drawn attention to study them in SSc where a similar profibrotic role is described. Studies have shown that elevated levels of OPN has been found to be significantly higher in skin biopsies and circulating plasma for both dcSSc and lcSSc patients when compared to healthy controls [143,144,145]. Using an murine bleomycin-induced dermal fibrosis model, Wu et al. demonstrated that OPN^-/-^ mice exhibit a reduced fibrotic and inflammatory response compared to WT counterparts [143]. Lesional skin biopsies revealed that OPN^-/-^ animals have a decrease in dermal thickness, a reduced concentration of αSMA-expressing myofibroblasts, and less Mac-3-positive macrophages in the dermal layer. Such mice also have limited TGFβ expression in areas of lesional skin compared to WT animals, which likely contributes to the attenuated fibrotic response.

## 8. Gla-Protein Family

Periostin (POSTN) and matrix Gla protein (MGP) are two vitamin K-dependent γ-carboxyglutamic acid-containing proteins that reside in the ECM, and together with 17 other members, make up the MCP family known as the Gla-family. The Gla-family of proteins are known primarily for their importance to bone metabolism and calcification, but recently POSTN and MGP have also been demonstrated to be involved in the processes of ECM remodeling, wound repair, and cancer [56,146,147,148,149]. In the context of SSc, emphasis has been placed on elucidating the role of POSTN, whereas MGP is much less understood for its role as an MCP than that of its inhibition of calcification. 

POSTN expression is consistently reported as being upregulated in SSc at varying stages of disease progression, and is detectable in serum and in biopsies of lesional skin, dermal, and lung primary fibroblasts [150,151,152,153]. Through analysis of serum POSTN, higher expression levels showed a positive correlation to both disease duration and severity of skin fibrosis [150]. Immunohistochemical staining for POSTN in SSc skin showed a concentrated localization in both the upper dermis and lower reticular layer where colocalization with αSMA-positive myofibroblasts was observed, indicating a potential association with the development of skin fibrosis [150]. Using an in vivo mouse model of bleomycin-induced scleroderma, knockout of POSTN (POSTN^-/-^ mice) provided substantial protection against dermal thickening by limiting uncontrolled collagen deposition compared to WT mice [152]. Moreover, skin sections of POSTN^-/-^ mice after bleomycin treatment contained fewer αSMA-positive cells compared to WT, suggesting that POSTN may be crucial for myofibroblast development in this model. Following up with in vitro experiments, isolated mouse dermal fibroblasts from POSTN^-/-^ mice were unable to sustain αSMA expression after TGFβ stimulation, compared to cultured fibroblasts from WT mice. Although an initial transient upregulation was observed, the enhanced αSMA expression was not upheld, suggesting that TGFβ requires the presence of POSTN for myofibroblast transformation. Lastly, immunohistochemical staining for POSTN in skin biopsy sections of scleroderma patients found POSTN expression to be entirely diffused throughout the dermis and subcutis layers [153]. As seen by confocal microscopy, POSTN was deposited within pockets of high αSMA concentration from these biopsy sections, in comparison to matched control samples.

MGP is a matrix-residing protein widely distributed in tissues including bone, kidney, lung, and cartilage, and is secreted by various cell types such as vascular smooth muscle cells and chondrocytes [154]. MGP is most notable for inhibiting vascular calcification through its five Gla-residues sequestering extracellular calcium ions and growth factors, such as bone morphogenetic proteins (BMP-2,4,7), in a vitamin K-dependent manner [155,156]. In SSc pathology, MGP is expressed in areas of dystrophic calcification, a frequently observed disease manifestation when deposits of calcified material occur in soft tissue [157]. Furthermore, it was found that MGP expression is upregulated in the skin of lcSSc patients with calcinosis compared to those without calcinosis [157]. A possible explanation for this observation may be that the gamma-carboxylation of MGP is required to prevent calcinosis by binding and inhibiting calcification-inducible factors. However, given that MGP has been recently described in tissue remodeling processes [146,149], further investigation is necessary to verify if a link exists between MGP expression and fibrosis in SSc.

## 9. Therapeutic Implications of MCPs in SSc

Extracellular localization, expression restricted to tissue remodeling processes, and strong association to disease progression are among the key characteristics of MCPs that offer inroads to be exploited in developing therapeutic tools and strategies. The clinical utility of MCPs are evolving beyond roles as merely informative biomarkers, which still remains an important exploratory route, but they are also being explored as drug delivery vehicles, therapeutic silencing targets, and components in biomaterials [158,159,160]. An informative review of MCPs and their burgeoning role as therapeutic targets in various disease contexts is referenced [149,158]. Herein, we summarize several recent examples of emerging therapeutic solutions that highlight the intersections between MCPs and SSc.

The CCN family has been a target of therapeutic interest since many of its studies have associated it to SSc more than any other MCP. Although several of the CCN members have contributed to fibrotic studies, CCN2 has had more success than any other member into transitioning clinically. A well-known human monoclonal neutralizing antibody inhibitor of CCN2, FG-3019, has been assessed in various disease models, including a model of murine Angiotensin-II-induced skin fibrosis [161]. A 2-week treatment with FG-3019 significantly reduced dermal thickness and collagen concentration versus control. FG-3019 treatment also reduced the fibrotic TGFβ pathway as evidenced by decreased phosphorylation of its downstream SMAD2 target. With a good outcome in a phase II trial (PRAISE, NCT01890265), FG-3019 is now currently proceeding into a phase III trial for idiopathic pulmonary fibrosis (NCT03955146), a frequent disease complication in SSc [162]. Pravastatin has recently been shown to also inhibit CCN2. Used as a specific inhibitor of the Rho/ROCK signaling pathway, pravastatin was found to impede CCN2 activation in ex vivo human intestinal tissue, which was further validated in rat models of intestinal fibrosis [163]. In fact, the direct downstream activation of CCN2 through the Rho/ROCK pathway by statin derivatives is a well noted observation [164]. Mechanistically, the inhibition of CCN2 mRNA and protein expression in pravastatin-treated rats limited deposition of type I collagen and fibronectin in a dose-dependent manner. The mounting evidence of CCN2 inhibition with repurposed drugs as an effective strategy for fibrotic management is encouraging because it provides a better understanding of the pharmacological mechanism of action of such treatments.

Although many more clinical studies are needed to prove the efficacy of targeting MCPs, promising therapeutic studies have provided evidence of indirectly lowering MCPs to affect SSc, implying cotargeting molecules for potential effective treatments. Fresolimumab is a TGFβ-neutralizing antibody that targets all of its mammalian isoforms and has been successful in a phase I clinical trial of early dcSSc patients (NCT01284322), reducing SSc disease biomarkers and improving skin symptoms [94]. Fresolimumab treatment downregulated profibrotic genes *CCN2*, *COL10A1*, and *SERPINE1* in skin biopsies, which also correlated with decreases in MRSS scores and dermal myofibroblast infiltration. Alongside MRSS scores, THBS-1 and -5 were used to evaluate the efficacy of fresolimumab for its rapid decline of TGFβ-regulated gene expressions, which demonstrated the capability of MCPs to serve as biomarkers in decision-making of tolerable drugs. Another recent study investigated the remedial properties of glycyrrhizin in a bleomycin mouse model of scleroderma. Treatment attributes included a blockage of key TGFβ transcription factors (SMAD3 and Ets1) along with a downregulation of THBS-1 in dermal fibroblasts, leading to significant amelioration of dermal fibrosis [165]. Targeting exosomes may also hold therapeutic potential to treating SSc, which would involve a mechanism implicating MCP expression Exosomes are nanovesicles composed of proteins and nucleic acids which have been shown to increase MCP expression in different fibrotic diseases, including SSc [166,167]. Compared to normal serum exosomes, isolated serum exosomes from SSc patients displayed higher levels of fibrotic microRNAs that were capable of increasing the expression of fibrotic genes, including CCN2 [168,169]. As we discover a larger number of MCP genes affected by exosomes in SSc, manipulating the contents, secretion, and delivery of such exosomes may prove to be effective in combating fibrosis from a therapeutic standpoint.

Beyond MCPs, several promising therapeutic interventions for managing SSc fibrosis have been reported but not yet validated in randomized controlled trials [170]. Drugs such as imatinib mesylate, pirfenidone, and rapamycin have shown antifibrotic potential in murine models of SSc skin and lung fibrosis, but a complete understanding is still lacking. Therefore, continued research and application of MCPs in sclerodermic models is wholly justified to efficiently develop therapeutic options that could be used independently or in combination of current treatments for effective options that impede and prevent fibrosis development.

## 10. Conclusions

The evidence has been promising to associate MCPs as essential contributors to the fibrotic pathophysiology in SSc. Each MCP family, albeit not every family member, has been shown to affect fibrotic processes in both primary and secondary roles by working in concert with profibrotic mechanisms such as TGFβ signaling, aberrant secretion of major ECM proteins (type I collagen and fibronectin), mechanotransduction, and myofibroblast differentiation. As the literature continues to expand and clarify many of the unaddressed mechanistic details of MCPs, parallels from related fibrotic pathologies may provide useful insights to translate into SSc [171]. This is especially true for the undercharacterized MCPs that lack direct studies investigating their roles in SSc or for proteins that currently serve a limited utility. MCPs have been emerging as reliable informative biomarkers in SSc since they are detectable in various forms of biological samples as well as holding their value in comparison to established metrics like the MRSS system. Future outlook of MCPs in SSc is promising with growing applications in clinical settings, as briefly highlighted in this review. Overall, research into the emerging roles of MCPs in SSc is an exciting endeavor owing to the breadth of known fibrotic functions that MCPs have in various organs, which will aid in defining their practical use in managing this vastly heterogenous disease.

## Figures and Tables

**Table 1:** Summary of major matricellular proteins (MCPs) in systemic sclerosis (SSc) fibrosis

MCP Family	MCP Family Members Upregulated in SSc	Bound Receptors	General Fibrotic Roles in SSc
**CCN [36,42,45,46]**	CCN1	Integrin (α_2_β_1_, α_6_β_1_, αIIbβ_3_, αDβ_2_, αMβ_2_, αvβ_1_, αvβ_3_, αvβ_5_), syndecan-4	• Both pro- and antifibrotic roles • CCN1 KO in mice limited type I collagen • Overexpression of CCN1 in SSc dermal fibroblasts downregulates *COL1A1* expression
	CCN2	Integrins (α_4_β_1_, α_5_β_1_, α_6_β_1_, αMβ_2_, αvβ_1_, αvβ_3_), LRP1, LRP6, syndecan-4, TrkA	• Expression induced by TGFβ-SMAD signaling and mechanotransduction mechanisms • Induces αSMA expression in SSc lung fibroblasts
	CCN3	Integrins (α_5_β_1_, α_6_β_1_, αvβ_1_, αvβ_5_)	• Overexpression in *Tsk1/+* mice downregulates fibrillin-1 and TGFβ/Wnt profibrotic genes
**THBS [36,47,48,49,50,51]**	THBS-1	Integrins (αvβ_3_, αIIbβ_3_, α_9_β_1_, α_6_β_1_, α_5_β_1_, α_4_β_1_, α_3_β_1,_ α_3_β_1_), CD36, CD47, CD148, LRP1, syndecan-3 and -4, calreticulin	• Endogenous activator of latent TGFβ • Enhances contractile activity of SSc fibroblasts by activation of MEK/ERK pathway
	THBS-2	Integrins (αvβ_3_, αIIbβ_3_, α_9_β_1_, α_6_β_1_, α_4_β_1_), CD36, CD47, LRP1, syndecan-4	• Silencing in SSc fibroblasts downregulates type I collagen synthesis
	THBS-4	Integrins (β_1_D, β_2_, β_3_)	• Supports collagen deposition (I, II, III, V) and displays proangiogenic effects through TGFβ-SMAD3 signaling
	THBS-5	Integrins (α_5_β_1_, αvβ_3_)	• Promotes aberrant secretion of collagen and fibronectin leading to matrix stiffening
**SPARC [52,53,54]**	SPARC	Integrin α_5_β_1_,TGF-β receptor, endoglin	• Inhibition in SSc dermal fibroblasts downregulates type I collagen and CCN2 • Blocking TGFβ-SMAD2 signaling limits profibrotic functions of SPARC in vitro
	FSTL1	Integrin β3	• Limited reports on fibrotic roles but evidence supports a proangiogenic role in SSc dermal endothelial cells
**TN [36,43,44]**	TN-C	Integrins (α_2_β_1_, α_5_β_1_, α7β_1_, α_8_β_1_, α_9_β_1_, αvβ_3_, αvβ_6_, αvβ_1_), Syndecan-4, Annexin II, EGFR, TLR4	• Mediates both profibrotic and inflammatory signaling by TLR4-dependent mechanism • KO mice limits infiltration of myofibroblasts, macrophages, and lymphocytes in pulmonary fibrosis
**SIBLING [36,55]**	OPN	Integrins (αvβ1, αvβ3, αvβ5, αvβ6, α5β1, α8β1, α9β1, α4β7, and α4β1) and CD44	• Regulates deposition of type I and IV collagen, • Regulates local TGFβ activity • Stimulates myofibroblast proliferation
**Gla-Family [56]**	POSTN	Integrins (αvβ3, αvβ_5_)	• Colocalizes with αSMA-positive expressing cells in lesional skin • Induces fibroblast differentiation to myofibroblasts • Promotes collagen deposition
	MGP	Unknown	• No direct studies linking MGP to fibrosis in SSc but may be implicated with calcinosis with SPARC

## Abbreviations

SSc	systemic sclerosis
dcSSc	diffuse cutaneous systemic sclerosis
lcSSc	limited cutaneous systemic sclerosis
ECM	extracellular matrix
αSMA	alpha-smooth muscle actin
MCP	matricellular protein
CCN	centralized coordination network
THBS	thrombospondin
SPARC	secreted protein acidic and rich in cysteine
TN	tenascin
SIBLING	Small Integrin-Binding Ligand N-Linked Glycoprotein
Gla	γ-carboxyglutamate
MMP	matrix metalloprotease
Tsk1/+	tight skin 1
MRSS	modified Rodnan skin score
HADC	histone deacetylase
EC	endothelial cell
WT	wild-type
TLR	toll-like receptor
OPN	osteopontin
POSTN	periostin
MGP	matrix Gla protein
